# Hepatitis C Virus in American Indian/Alaskan Native and Aboriginal Peoples of North America

**DOI:** 10.3390/v4123912

**Published:** 2012-12-19

**Authors:** Julia D. Rempel, Julia Uhanova

**Affiliations:** 1 Section of Hepatology, Department of Medicine, Department of Immunology, University of Manitoba, 804D-715 McDermot Ave, Winnipeg, MB, USA; 2 Section of Hepatology, Department of Medicine, University of Manitoba, 803B-715 McDermot Ave, Winnipeg, MB, USA; E-Mail: uhanovaj@cc.umanitoba.ca

**Keywords:** hepatitis C virus, AI/AN, Aboriginal, female, HIV, trauma

## Abstract

Liver diseases, such as hepatitis C virus (HCV), are “broken spirit” diseases. The prevalence of HCV infection for American Indian/Alaskan Native (AI/AN) in the United States and Canadian Aboriginals varies; nonetheless, incidence rates of newly diagnosed HCV infection are typically higher relative to non-indigenous people. For AI/AN and Aboriginal peoples risk factors for the diagnosis of HCV can reflect that of the general population: predominately male, a history of injection drug use, in midlife years, with a connection with urban centers. However, the face of the indigenous HCV infected individual is becoming increasingly female and younger compared to non-indigenous counterparts. Epidemiology studies indicate that more effective clearance of acute HCV infection can occur for select Aboriginal populations, a phenomenon which may be linked to unique immune characteristics. For individuals progressing to chronic HCV infection treatment outcomes are comparable to other racial cohorts. Disease progression, however, is propelled by elevated rates of co-morbidities including type 2 diabetes and alcohol use, along with human immunodeficiency virus (HIV) co-infection relative to non-indigenous patients. Historical and personal trauma has a major role in the participation of high risk behaviors and associated diseases. Although emerging treatments provide hope, combating HCV related morbidity and mortality will require interventions that address the etiology of broken spirit diseases.

## 1. Introduction

There are major health disparities between indigenous and non-indigenous peoples in the United States (US) and Canada. In the US, American Indian (AI) or Alaskan Native (AN) represent 1.2% of the population (see Table 1 for terms) [1]. Within Canada, Aboriginal peoples account for 3.7% of the population consisting of 4% Inuit, 60% First Nation (FN) and 36% Métis (36%), with 2% being undeclared in the 2006 census [2]. Despite the simplicity in this representation, these populations contain a large variety of cultures and languages.

**Table 1:** American Indian/Alaskan Native and Aboriginal peoples of North America

Country	Term	Definition *	% Population
Global	Indigenous	Peoples with pre-historical ties to a land, prior to colonization.	370 million, world wide [3]
United States (US)	American Indian/Alaskan Native (AI/AN)	Approximately 561 tribes are recognized by the US government.	1.2% of US population (2011) [4]
	Alaskan Native	Peoples indigenous to Alaska. Alaskan Natives include Eskimos such as the Yupik, Inupiat and Aleut. The term Eskimo is used since it is inclusive of Inuit and non-Inuit peoples. In addition to the Eskimo, there are 11 Athabaskan tribes or language groups.	14.9% of Alaska’s population (2011) [5]
	American Indian	Peoples indigenous to the continental US, includes Athabaskan and a wide variety of other tribal groups	Approximately 1.2% of US population [4]
Canada	Aboriginal or First Peoples	All individuals of indigenous ancestry within Canada	3.7% of Canada (2011) [2,6]
	Inuit	Peoples indigenous to the four northern regions of Canada: Nunatsiavut (Labrador), Nunavik (northern Quebec), Nunavut, and the Inuvialuit Settlement Region in the Northwest Territories. Formerly referred to as Eskimo. Also found in Alaska, Greenland and Russia. In Alaska they are included in the designation Alaskan Natives.	4% of Aboriginals [2,7]
	First Nation (FN)	Indigenous peoples with and without Indian Registered Status according to Indian Act of Canada (613 bands). Includes a large number of Athabaskan and as well as other tribal groups. Also referred to as Indian, although this term has fallen into disuse since the 1980s as “Indian” is linguistically incorrect. Nonetheless, “First Nation” is not a legal term, whereas “Indian” is. Akin to the US term American Indian.	60% of Aboriginals [2]
	Métis	Historically Métis Nation ancestry implies “children of the fur trade”. However, broadly this term has also has come to include self-identified Aboriginal individuals of mixed indigenous ancestry who are not considered Inuit or First Nation. Note: Inuit and First Nation individuals can also be of mixed ancestry.	36% of Aboriginals [2,8]
Greenland	Inuit	Ancestors migrated from Canadian North.	89% of 57,695 [9]

* Definitions are for the purpose of this review. However, these definitions are not necessarily universally agreed upon.

## 2. Liver Disease in Indigenous Peoples of North America

US AI/AN and Canadian Aboriginal peoples have a higher prevalence of liver disease than other peoples. US statistics indicate that chronic liver disease is the 5th leading cause of mortality for AI/AN peoples. For all races combined it is 12th [10]. Comparably, liver disease is the 13th cause of mortality in Canada. Although Canadian health statistics are not assessed according to race, British Columbia (BC) Health Statistics reported that liver disease was the 8th leading cause of death for Aboriginal peoples but 23rd for non-Aboriginal people [11]. This disparity is likely to be the case across Canada.

Multiple dynamics contribute to these racial differences including an enhanced susceptibility to autoimmune liver disease such as autoimmune hepatitis and primary biliary cirrhosis [12,13,14]. In addition, liver diseases comprise of “broken spirit” diseases. These encompass alcoholic induced liver disease, hepatitis C virus (HCV) and hepatitis B virus (HBV), along with the rise of non-alcoholic induced fatty liver disease (NAFLD) and non-alcoholic steatohepatitis (NASH) [13,15]. This review focuses on HCV infection in AI/AN and Aboriginal peoples of North America.

## 3. Impact of HCV on Indigenous Peoples Globally

Globally, it is estimated that 170 million people are HCV infected. In colonized countries, the prevalence of HCV infection in indigenous populations tends to be higher than non-indigenous populations. To illustrate, Australian national surveillance between 2004 and 2006 found that 138/100000 Aborigines vs. 38/100000 of the remainder population were positive for antibodies against HCV [16]. South America indigenous peoples, that are less urbanized, are an exception [17]. Here, prevalence rates were reported to be 0-1.7% [18,19,20,21]. However, these rates may be on the rise as external influences grow. For the Yukpa and Bari natives of Venezuela, for instance, prevalence rates have increased from 0 to 2.2% between 1994-2002 [22,23].

In the US and Canada, an estimated 1.6% and 0.8% of individuals respectively are HCV infected [24]. The proportion of indigenous peoples that are HCV infected is largely unknown. In northern regions, few population based prevalence studies have been performed. HCV infection in AI/AN populations of Alaska were typically low (1999 and 2001) in rural regions (1%) but higher in urban centers (15%) [25]. Prevalence rates for two Canadian Inuit communities have been described at 1.0% (n=720) and 18% (n=190) [25]. The Inuit of Greenland (either living in Greenland or Denmark) have HCV seropositivities ranging between 0% and 1.5% [26,27]. In a FN community further south, HCV seropositivity was observed to be 2.2% (n=7/315) [28]. Population studies in other regions or centers are generally lacking [25].

Clinic based studies frequently find a high prevalence of HCV infection in AI/AN and Aboriginal populations. A Nebraskan general admissions clinical survey of patients or their family members (n=249) found a HCV antibody prevalence of 8.1% and 18.3% for females and males respectively [29]. Between 2000-2002, Indian Health Service (IHS) clinics in Arizona (n=1496) describe a prevalence of 6.9%; whereas, in California (2002-2003) a 24.1% prevalence was observed in a study of 344 individuals [30]. Manitoban provincial laboratories found the HCV seroprevalence (per 100,000 persons) to be 2.4 greater for FN, than non-FN, individuals [31].

Incidence studies focus on “acute” or newly diagnosed HCV infections. In the US, newly diagnosed HCV infections significantly declined for all racial groups from 1999 to 2009 [32,33]. The particularly high incidence levels at the turn of the century may represent the initiation of HCV testing and reporting throughout the US. Thus, these earlier studies may reflect a population “backlog” as opposed to an actual decrease in the acquisition of new HCV infections. These early reports indicate (2001) indicated that the incidence of HCV infection in Native Americans was six-fold greater than the general population. Over time, rates have become more comparable; such that by 2009, rates for AI/AN, Caucasian and African American populations were observed to be 0.41, 0.23 and 0.11 per 100,000 persons respectively [33]. However, a 2011 publication determined that the number of hospitalizations due to acute HCV infection within IHS clinics was escalating signally that the rate of HCV acquisition has not waned in these areas [34].

In Canada, 15.2% newly acquired infections could be attributed to Aboriginals and 82.7% to non-Aboriginal from 1999 to 2004 over six health regions in BC, Alberta, Manitoba, Ontario and New Brunswick [35]. This corresponded to a HCV infection incidence of 18.9/100, 000 (95% CI 15.5–23.1) and 2.8 (95% CI 2.6–3.1) respectively for Aboriginal and non-Aboriginal populations [35]. This is also observed in a separate Manitoban report where 112.2 FN compared to 45.5 non-FN peoples (per 100,000 persons) accounted for new HCV infection diagnoses between 2000 and 2002 [36]. Taken together, incidence studies indicate that AI/AN and Aboriginal peoples suffer an increasingly higher burden of HCV infection than other populations. 

## 4. Risk Factors for the Acquisition of HCV Infection

The prevailing reason for HCV infection is injection drug use (IDU). In IDU and other high risk groups, HCV antibody seropositivity can range from 8.1% to 63.8% [37,38]. Potential interactions with drug cultures have a major impact on the rate of HCV seropositivity as referenced above. The association of HCV infection with urban, versus rural, settings is apparent in northern regions [31,39,40]. In Siberia, for example, two serosurveys reported a prevalence of anti-HCV antibody ranging from 0.93% to 1.4% [41,42]. However, a third urban study found that the anti-HCV prevalence ranged from 2.1% for blood donors to 6.4% for medical students [43]. As previously mentioned, parallel findings are reported in Alaska and other circumpolar regions [31,39,40]. In northern Manitoban, there are similar rates of HCV infection between FN and non-FN individuals. Southern rural areas, in contrast, have a relative rate (RR) for infection for FN/non-FN of 4.1-4.6, whereas in Winnipeg the RR is 7.1 [36]. As detailed by Wylie *et al.*, multivariate analysis determined a recent move to the city, but not Aboriginal ethnicity, was an indicator for HCV and HBV infection [44]. This suggests that the initiation of high risk activities that fosters these diseases occurs once individuals move to urban centers.

Within high risk cohorts, similar HCV seroprevalences between FN and non-FN, or self-identified Aboriginal and non-Aboriginal, cohorts have been observed in a number of studies [37,44,45,46,47]. Moses *et al.* reported comparable seroprevalences between FN (19.4%), Métis (22.3%) and non-Aboriginal (14.4%) individuals [45]. The relationship of HCV infection and behaviors can also be observed for prison and supervised safer injection facilities [46,47]. IDU as a risk factor for HCV infection often corresponds to being male, middle age and participation in activities such as alcoholism, HCV positive partners or tattoos [29,48]. Transfusions prior to the early 1990’s are another risk factor. These are also characteristics of HCV infection in AI/AN populations as reported in Nebraska and Alaska [29,40,48].

Disconcertingly, the face of the AI and Aboriginal HCV infected patient is changing. In many urban centers, the face of the FN and Métis HCV infected patient is increasingly female [31,35,38]. In BC west coast and central interior, HCV infection was 1.7-1.9 times greater for women than men [49,50]. In central Canada the age-adjusted incidence for female gender for FN compared to non-FN was 4.1 [36]. The face of the HCV infected Aboriginal is also becoming increasingly younger. Evaluation of the HCV demographics in six health regions throughout Canada indicated that Aboriginals were diagnosed with HCV infection five years earlier than non-Aboriginals [35]. In Manitoban FN populations HCV infection occurred approximately seven years younger than non-FN counterparts [36]. Thus, HCV infection appears to be an increasing weight on Aboriginal young women.

In addition to be being female and younger, HCV infected Aboriginals were less educated and more often involved in the sex trade [51]. Sexual intercourse is not generally considered a mode of transmission for HCV infection. However, the number of sex partners associates with the risk for HCV transmission [52]. This risk can be augmented by the presence of pre-existing human immunodeficiency virus (HIV) infection in the absence of drug use, discussed further below [53]. The emerging interaction of IDU and sex work with newly acquired HCV infection speaks to social pressures and determinants present for young Aboriginal women in this disease’s current etiology [31,35,38]. Although these are predominately Canadian findings, it is possible that these shifts are occurring in the US as well.

## 5. Rates of Chronicity

Acute HCV infection commonly progresses to chronicity in 65-85% of cases [54]. Comparable rates of chronicity have been observed for certain indigenous peoples, but populations of an enhanced resistance to chronicity also exist. In northern AN populations chronicity rates are analogous to non-indigenous peoples [40]. In contrast, HCV RNA was generally not observed in HCV antibody positive individuals in one northern Canadian Inuit community [25]. Likewise, HCV chronicity was below 6% for Greenland Inuit peoples [26,27]. 

In the Nebraskan clinical study, 75% of the HCV seropositive individuals were also HCV RNA positive, indicating that three quarters of acutely infected individuals developed a chronic infection [29]. Conversely, significantly reduced rates of progression to chronic HCV infection have been detected for indigenous peoples in the west coast and plains regions of Canada. In IDU cohorts of BC, self-identified Aboriginals that were HCV antibody positive demonstrated HCV RNA negativity at an odds ratio of 2.9 (95% CI 2.0 to 4.3; p<0.001) relative to non-Aboriginals; strongly suggestive of enhanced spontaneous clearance [55]. A Manitoban FN community survey found that of the 2.2% anti-HCV positive individuals, none tested positive for HCV-RNA [28]. The corresponding provincial testing center further observed a reduced HCV chronicity in FN relative to non-FN patients, despite HCV infection being suspected as the cause of liver dysfunction in these patients [56]. For other US AI and Canadian Aboriginal peoples the rate of progression to chronic HCV infection has not been investigated.

Analyses of re-infection rates in IDU cohorts have been inconclusive as to whether spontaneous clearance of HCV infection is protective against subsequent re-infection [57,58,59,60,61]. It remains to be determined if re-infection rates differ between AI/AN and Aboriginal population relative to non-indigenous counterparts.

A variety of elements could contribute to improved rates of self-limited infection in certain Aboriginal populations including individual age at time of exposure and viral factors. Upon pediatric exposure spontaneous clearance of HCV infection can occur in 30% -50% of cases, although for vertical transmission rates appear considerably lower (9%) [62,63,64]. A younger age of exposure is a not likely a critical factor in the enhanced clearance observed within older Aboriginal cohorts, as the acquisition of HCV infection is associated moving into an urban center [44]. However, with young women increasingly affected by HCV infection, pediatric diagnoses may rise for indigenous populations. Due to the strong association of HCV infection with IDU limited differences in dose and route would account for racial differences in enhanced clearance of acute HCV infection. Yet again, it is unknown if emerging transmission routes will alter the potential for self-limited HCV infection, especially in HIV co-infection individuals (see below).

Another possibility is that physiological characteristics unique to AI/AN and Aboriginal peoples may support an enhanced capacity to spontaneously clear HCV infection. HCV chronicity has been associated with host genetics and viral impairment of host immunity. Thus, we have been investigating whether differences in immunity between Aboriginal and non-Aboriginal populations to allow for more effective clearance of acute HCV infection.

Interleukin (IL)-10 is an anti-inflammatory cytokine that can inhibit the T cell interferon (IFN)-γ production that is associated with spontaneous HCV clearance [65]. The ability of HCV core protein upon initial viral exposure to trigger immune cells such as monocytes (a population of cells in peripheral blood mononuclear cells, PBMC) to produce IL-10 is considered critical in this process [66]. In addition, individuals with a reduced genetic tendency to produce IL-10 appear less likely to progress to chronic infection [67,68,69]. Genetic studies by our lab and others indicate that Manitoban FN and Métis populations have a reduced genetic tendency to produce IL-10 compared to non-Aboriginals [70,71]. Moreover, we found that PBMC from virally-naïve FN individuals produced significantly less IL-10 in response to HCV core protein than cells isolated from non-FN individuals, suggesting that FN cells were not as susceptible to immune deregulation upon first encounter with the virus [70,72]. 

We further evaluated whether differences in killer Ig like receptors (KIR) gene polymorphisms might influence spontaneous clearance in Aboriginals. KIRs are involved in CD8 T cell and natural killer cell cytotoxic immunity against viruses. Unique KIR gene profiles consisting of a greater presence of activatory genes have been published for patients with self-limited or benign HCV infection outcomes relative to patients with deteriorated disease courses [73,74]. The disparities in KIR gene profiles of virally naïve Aboriginals as compared to non-Aboriginals paralleled these disparities reported for better as compared to worse outcomes for HCV infection, suggesting the further involvement of biological processes in racial trends of HCV chronicity [72]. Coincidently, the unique KIR gene profile and genotypes of Manitoban Aboriginals were similar to other KIR analyses performed within indigenous peoples throughout the Americas; whereas, data from Manitoban Caucasians reflected that of Caucasian globally [72]. This might be accounted for by a common ancestry or an abrupt immune selection such as the “old world” disease epidemics that swept through indigenous communities [75,76,77,78]. A more aggressive immunity could have selected for survival under such harsh conditions, resulting in these genetic profiles being passed to the next generations. We are continuing to examine cytokine and KIR profiles in Aboriginal and Caucasian patients with self-limited or chronic HCV infection in an attempt to determine biological factors contributing to HCV chronicity in Manitoban Aboriginals. Taken together, within certain Aboriginal populations specific pro-inflammatory or activatory immune characteristics may be linked to more effective clearance of acute HCV infection. The contribution of other variables should also be considered in understanding this phenomenon. 

## 6. Treatment Outcomes

Despite an enhanced clearance of acute HCV infection in various Aboriginal populations, the high incidence of HCV infections has a significant impact on the health of AI/AN and Aboriginal peoples. Thus, it is important to understand the utility of treatment in these populations.

The backbone for HCV treatment is pegylated IFN (peg-IFN) and ribavirin (RBV). Treatment outcomes are influenced by viral genotype and load. This therapy is effective for up to 80% genotype 2 and 3 infections and for 40-50% of genotype 1 HCV infections [79,80,81]. The distribution of HCV genotypes in indigenous peoples tends to parallel non-indigenous groups in North America, with genotype 1 being dominant, then 2 and 3, with genotypes 4, 5 and 6 being rarer [40,82,83]. 

Race and other host factors including age, body mass and co-morbidities also influence therapeutic responses. The impact of race has been well documented for African Americans where sustained viral response (SVR) rates for genotype 1 infections are about half of those for Caucasians [84]. In assessing therapeutic responses for Aboriginals, Cooper *et al.* found a similar percent of SVR between Aboriginal and non-Aboriginal people infected with genotype 1 virus [85]. Comparable findings have been observed by our group (Minuk, *et al.*, unpublished data). Nonetheless, there may be some ethnic advantage as Cooper *et al.* reported a reduced tendency to relapse in Aboriginals (13%) than non-Aboriginals (31%, p= 0.013) [85].

With the recent introduction of protease inhibitors telaprevir or boceprevir along with peg-IFN/RBV, triple therapy schedules have markedly enhanced the ability to eliminate genotype 1 infections [86]. Protease inhibitors are able to augment viral clearance 1.5-3.8 fold even in the case of previous treatment failures. Studies examining triple therapy in AI/AN or Aboriginal populations have yet to be published. However, these treatment components have a variety of side effects. The potential of peg-IFN to cause adverse events has been well documented and can include flu like symptoms, fatigue, thrombocytopenia and psychiatric side effects [87,88]. Adverse events due to protease inhibitors are also becoming apparent. It is unknown if adverse effects to components of HCV therapy differ between indigenous and non-indigenous populations.

In an effort to alleviate additional health complications that can be caused by treatment, numerous investigations attempting to predict therapeutic responses have been performed. One approach has been to examine the ability of genetic polymorphisms, including IL-10, to forecast the ability to achieve a SVR [89]. IL-28B polymorphisms have surfaced as being able to consistently predict treatment outcomes. The rs12979860 CC genotype was strongly associated with successful peg-IFN/RBV treatment for genotype 1 infection compared to the CT/TT for African American, Asian, Caucasian, and Hispanic patient cohorts [90,91]. In response to triple therapy, rs12979860 CC still has some predictive value, but it is lost if patients achieve a rapid viral response into 4 weeks of therapy [92]. We are currently evaluating whether IL-28B has the same utility in predicting treatment outcomes in Aboriginal HCV infected individuals.

## 7. Disease Course

Despite decreasing incidences of newly diagnosed HCV infection, the mortality attributed to HCV infection has been steadily increasing since 1999. By 2007, HCV surpassed HIV as a cause for mortality in the US [93,94]. This dichotomy is likely due to the capacity for HCV infection to lie dormant in the body for decades prior to manifestation. After 20-30 years of infection, 2-20% of individuals will develop cirrhosis, which can progress to hepatocellular carcinoma or liver decompensation [24]. Critical risk factors for progression to liver failure include older age, male sex, alcohol use and co-current conditions such as diabetes, steatosis, HBV or HIV infection [95,96,97,98].

The natural history of HCV infection in AI/AN or Aboriginal populations has not been well documented. In Manitoba, co-morbidities linked with a more rapid progression of HCV induced liver disease were more prevalent in FN, versus non-FN, HCV infected patients [36]. FN patients were more likely to be burdened with alcohol abuse (2-fold), diabetes (1.5 fold), and HIV infection (1.9 fold) relative to non-FN counterparts. Although the percentage of decompensated liver disease was similar between ethnic cohorts, FN patients died 12 years younger than the non-FN patients [36]. Thus, the heightened presence of risk factors for disease progression appeared to further impact the life expectancy of FN HCV infected individuals [99]. Independent of therapeutic outcomes, a history of alcohol use was the greatest indicator of end-stage liver disease and liver-related mortality in HCV infected AN patients [100]. However, the full impact of the rise of HIV co-infection in young indigenous population has yet to be realized.

## 8. HIV Co-infection

The rate of HCV and HIV infections varies within IDU populations. Typical results are reflected in a study by Pilon *et al.* where the prevalence of HCV infection was 61%, with HIV infection accounting for 10% of the study cohort (n=407) [101]. Within HCV infected IDU cohorts 1/10 are estimated to be co-infected with HIV [38]. However, the majority of HIV positive individuals that inject drugs tend to be HCV co-infected [38,101].

The greater incidence of newly diagnosed HCV infections in AI/AN and Aboriginal populations also signals an increase in affiliated diseases such as HIV infections [34]. Although rates of HIV infection appear comparable between AI/AN and other races in the US, in Canada street involved Aboriginal people have a higher incidence of HIV infection than non-Aboriginal cohorts [44,102]. HIV seroconversion can occur at twice the rate for Aboriginal relative to non-Aboriginal IDUs (p<0.001) [103,104]. In central Canadian IDU and street populations, HIV independently associated with Aboriginal ethnicity following multivariant analysis, even though HCV infection did not [44]. 

As result, there is an alarmingly high rate of HCV/HIV co-infection in street involved Aboriginals throughout Canada [105,106,107,108]. In BC, these rates are particularly startling with HCV co-infection being 7 times higher in HIV infected Aboriginals than in HIV infected non-Aboriginals (p<0.001) [108]. Notably, a corresponding dynamic was apparent for females versus males (18% vs. 3%, p<0.001) [108]. Aboriginals on antiretroviral therapy were also more likely female (22% vs. 10%; p</0.001) and HCV co-infected (65% vs. 27%; p<0.001) [106]. HCV co-infection has a strong role in mediating HIV mortality as liver failure is the second leading cause of death in co-infected individuals [109]. HCV co-infection may contribute to the substantially faster progression of HIV infection in AI/AN and Aboriginal populations, including a shorter time from diagnoses to the onset of AIDS than other races [110,111]. In addition, even with treatment AI/AN and Aboriginal HIV infected individuals have a markedly faster progression to HIV related mortality than non-indigenous counterparts [102,112,113]. 

## 9. Etiology of IDU

AI/AN and Aboriginal peoples are over-represented among individuals who inject drugs or participate in high risk behaviors [46,104,114]. Factors that drive the participation in self-inflicted behaviors and the participation of these behaviors in the etiology of broken spirit diseases should be a concern for health policy advisors. It is known that illicit drug use is connected with childhood trauma [115,116,117]. The destructive effect of personal trauma is a key contributor to the participation of girls and young women in the sex trade [118], and childhood sexual assault is a major factor in driving young people, particularly women, to the streets resulting in exposure to HCV and HIV [49].

It is for these reasons, that a discussion on indigenous peoples in North America and diseases that are nurtured by trauma related behaviors, like HCV infection, needs to consider the history of colonization, including the impact of Indian policies, US Indian boarding schools and Canadian residential schools [114,119,120]. Aboriginal children experienced extensive psychological, sexual, physical and emotional abuses within those systems [121,122]. It is increasingly appreciated that the resulting trauma suffered by an entire generation of children has rippled through the subsequent generations [123]. Indeed, a Vancouver study found that HCV infection positively correlated with one parent having been in residential school [50]. An independent study in Saskatoon found that 38% of IDU attended residential school and 71% had a parent or grandparent that attended residential school [51]. The difference in these numbers may reflect the passing of the older IDU generation or may suggest that abuse from a stranger is more devastating than the ripple effect into the next generation. With the influence of residential school taken out of the calculation, Aboriginal IDU were still more likely to have experienced the death of, or permanent separation, from a parent than non-Aboriginal counterparts [51]. Thus, when considering preventions and interventions for IDUs and HCV infected patients, it is essential that issues concerning historical and personal trauma are addressed.

## 10. Financial Costs

The incidence of HCV infection in the US AI/AN and Canadian Aboriginal populations is 2-7 times greater than the general population. In IHS facilities, there was a four-fold increase in the number of hospitalizations due to acute HCV infection from 1995 to 2007. Moreover, these indigenous patients are testing positive for HCV infection in their 40’s or younger [29,40]. These events forecast the need for future medical interventions due to chronic HCV infection, cirrhosis, hepatocellular carcinoma and end stage liver disease [34]. It is expected that within decades the prevalence and cost of treating HCV infection morbidities will dramatically increase [124].

US estimates predict that HCV infection will generate direct medical costs of $10.7 (USD) billion between 2010 and 2019 [125]. Increases in HCV related hospitalizations are further anticipated to result in a substantial strain on the limited resources of the IHS [34]. In Canada, the treatment of HCV infection due to IDU is estimated to cost four billion dollars (CAD) by 2026 [37]. These estimates do not take into consideration indirect costs, such as losses to the labor force. These costs to North American economies could exceed direct costs seven fold [125] even before being compounded by the high rate of co-morbidities, including HIV co-infection [126]. Although new therapeutics promise hope for select patient populations, addressing risk factors for HCV infection is vital in lowering HCV-related morbidity and expenditures [35]. Investing in prevention strategies now would ease the future financial burden of HCV on our nations.

## 11. Summary

US AI/AN and Canadian Aboriginal peoples have a wide range of cultural practices and languages. Nonetheless, greater incidences of newly diagnosed HCV infection are observed. Despite lower rates of chronicity in certain Aboriginal populations, higher rates of co-morbidities such as diabetes, alcohol use and HIV infection contribute to a greatly reduced life expectancy of HCV infected indigenous populations. The diagnosis of HCV infection within indigenous peoples is associated with a history or current IDU. However, the face of the Canadian Aboriginal HCV infected population is shifting from middle aged men to younger women, a change that may also be occurring in the US. Although new therapies for HCV infection offer hope, prevention should be a priority. In prevention and treatment, it is necessary that historical and personal trauma be addressed as they are crucial in the etiology of high risk behaviors that drive HCV infection and other broken spirit diseases. 
